# The anti-inflammatory properties of vinpocetine mediates its therapeutic potential in management of atherosclerosis

**DOI:** 10.1186/s12950-024-00394-x

**Published:** 2024-06-10

**Authors:** Abdullah A. Alshehri, Hayder M. Al-kuraishy, Ali I. Al-Gareeb, Sabrean F. Jawad, Wael Y. Khawagi, Athanasios Alexiou, Marios Papadakis, Abdullah A Assiri, Heba Elhadad, Gaber El-Saber Batiha

**Affiliations:** 1 Department of Clinical Pharmacy, College of Pharmacy, Taif University, Al Huwaya, Taif, Saudi Arabia; 2 Department of Clinical Pharmacology and Medicine, College of Medicine, Al-Mustansiriya University, Baghdad, Iraq; 3 Jabir ibn Hayyan Medical University, PO.Box13, Al-Ameer Qu./Najaf, Iraq; 4 Department of Pharmacy, Al-Mustaqbal University College, Hillah, Babylon, 51001 Iraq; 5 Department of Science and Engineering, Novel Global Community Educational Foundation, Hebersham, NSW 2770 Australia; 6 AFNP Med, Wien, 1030 Austria; 7 Department of Surgery II, University Hospital Witten-Herdecke, Universityof Witten-Herdecke, Heusnerstrasse 40, 42283 Wuppertal, Germany; 8 Department of Clinical Pharmacy, College of Pharmacy, King Khalid University Abha, Abha, Saudi Arabia; 9 Department of Parasitology, Medical Research Institute, Alexandria University, Alexandria, Egypt; 10 Department of Pharmacology and Therapeutics, Faculty of Veterinary Medicine, Damanhour University, Damanhour, AlBeheira, 22511 Egypt; 11 University Centre for Research & Development, Chandigarh University, Chandigarh-Ludhiana Highway, Mohali, Punjab, India; 12 Department of Research & Development, Funogen, Athens, 11741 Greece

**Keywords:** Atherosclerosis, Endothelial dysfunction, Vinpocetine, Pro-inflammatory cytokines, Reactive oxygen species

## Abstract

Atherosclerosis (AS) formation is enhanced by different mechanisms including cytokine generation, vascular smooth muscle cell proliferation, and migration. One of the recent treatments towards endothelial dysfunction and AS is Vinpocetine (VPN). VPN is a potent inhibitor of phosphodiesterase enzyme 1 (PDE-1) and has anti-inflammatory and antioxidant effects through inhibition the expression of nuclear factor kappa B (NF-κB). VPN has been shown to be effective against the development and progression of AS. However, the underlying molecular mechanism was not fully clarified. Consequently, objective of the present review was to discuss the mechanistic role of VPN in the pathogenesis AS. Most of pro-inflammatory cytokines that released from macrophages are inhibited by action of VPN through NF-κB-dependent mechanism. VPN blocks monocyte adhesion and migration by constraining the expression and action of pro-inflammatory cytokines. As well, VPN is effective in reducing of oxidative stress a cornerstone in the pathogenesis of AS through inhibition of NF-κB and PDE1. VPN promotes plaque stability and prevents the erosion and rupture of atherosclerotic plaque. In conclusion, VPN through mitigation of inflammatory and oxidative stress, and improvement of plaque stability effects could be effective agent in the management of AS.

## Introduction

**Fig. 1:**
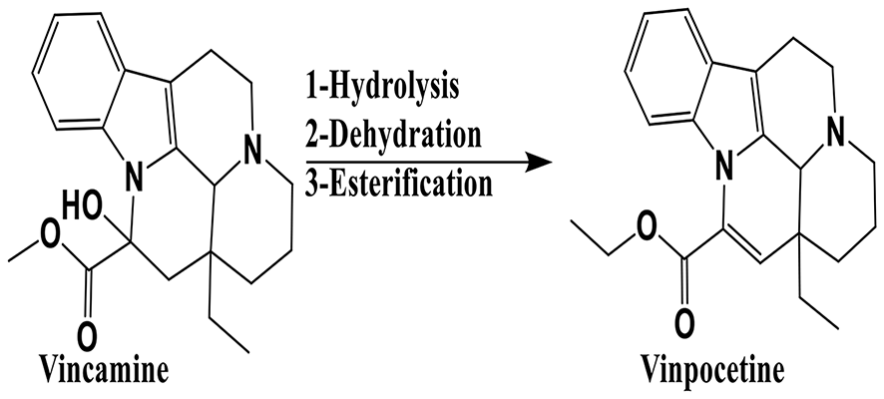
Semisynthesis of vinpocetine from natural alkaloid vincamine

Vinpocetine (VPN) is an ethyl apovincaminate derived from vinca alkaloid vincamine (Fig. 1) which extracted from *Vocanga Africana* seeds and Vinca minor leaves [1].

VPN was discovered in 1978 in Hungary, and have been extensively used in different cerebrovascular disorders [2]. VPN was used in the management of dementia and stroke in European and Asian countries, though it was not approved for therapeutic use in USA [3]. Despite of the extensive use of VPN as nootropic and dietary supplement, it was not approved by FDA. VPN was first used in 1978 in treating of dementia, stroke, and memory disorders [4].

The mechanism of VPN is related to inhibition of sodium channel, reduction of calcium influx and antioxidant effects [5]. VPN is regarded as potent inhibitor of phosphodiesterase enzyme 1 (PDE-1). VPN has an anti-inflammatory effect by inhibiting the expression of nuclear factor kappa B (NF-κB) through stabilization of IκB which is an inhibitor of NF-κB. Moreover, VPN have anti-platelet activity, thereby; improves brain blood flow and brain metabolism [6].

Currently, VPN is also available in the market as a dietary supplement to enhance cognition and memory. Due to its excellent safety profile, increasing efforts have been put into exploring the novel therapeutic effects and mechanism of actions of VPN in various cell types and disease models. Recent studies have revealed a number of novel functions of VPN, including anti-inflammation, antagonizing injury-induced vascular remodeling, and high-fat-diet-induced atherosclerosis (AS) as well as attenuating pathological cardiac remodeling. These novel findings may simplify the repositioning of VPN for preventing or treating relevant disorders in humans [7].

Prolonged use of VPN is associated with the development of certain adverse effects including hypotension, tachycardia, dizziness, dry mouth, nausea, heartburn and flushing [8]. To date, there have been no reports of significant side effects, toxicity, or contraindications at therapeutic doses of VPN on the cardiovascular system; therefore it is an interesting compound to explore novel therapeutic applications. A recent study shown a cardioprotective effect of VPN in a rat myocardial infarction model induced by acute treatment with isoproterenol. Isoproterenol-induced cardiomyopathy in rat is reflected by increased serum markers of myocardial infarction such as serum creatine kinase-MB, lactate dehydrogenase, glutamic oxaloacetic transaminase, and Troponin-T, as well as histopathological features of myocardial infarction such as myocardial necrosis, edema, infiltration of macrophages and lymphocytes. The cardiac damage induced by isoproterenol seemed to involve reactive oxygen species (ROS) and VPN treatment increased the activity of a number of antioxidant enzymes [9]. It has been shown that chronic Angiotensin II infusion induced cardiac hypertrophy and cardiac fibrosis is noticeably reduced by systemic administration of VPN. Furthermore, in isolated adult mouse cardiomyocytes, VPN inhibited Angiotensin II-stimulated cardiomyocytes hypertrophic growth. In cultured cardiac fibroblasts, VPN inhibits fibroblast activation and matrix gene expression, such as smooth muscle alpha-actin, type I collagen and fibronectin [10].

VPN has a specific pharmacokinetic profile; the effective therapeutic dosage of VPN is 5–10 mg [11]. VPN half-life is 1–2 h; it highly absorbed from intestine with 56.6% bioavailability, peak plasma level is reached after one hr of oral administration, highly distributed, cross blood brain barrier (BBB), metabolized by liver and excreted by urine [12]. It has been reported that VPN was effective against the development and progression of atherosclerosis (AS) [7]. Though, the underlying molecular mechanism was not fully clarified. Consequently, objective of the present narrative review was to clarify the mechanistic role of VPN in AS.

## Atherosclerosis overview

AS is a vascular disease characterized by thickening of the intimal layer of arteries and accumulation of fat. Fatty material is located in the central core of the plaque, covered by fibrous cap. The term, atherosclerosis consists of two parts; atherosis (accumulation of fat accompanied by several macrophages) and sclerosis (fibrosis layer comprising smooth muscle cells [SMC], leukocyte, and connective tissue) [13]. AS is an advanced disease impedes blood flow causing tissue ischemia predominantly in the brain and heart [14]. AS complications such as peripheral vascular disease, stroke and myocardial infarction are the major causes of mortality. AS process may be started in childhood but demonstrated clinically in the middle age and later [15]. Rupture of atherosclerotic plaques and associated thromboembolic disorders are the main reason for cardiovascular complications [16]. The underlying associated pathological conditions connected with AS progression are inflammation, oxidative stress, endothelial dysfunction, apoptosis, vascular proliferation, matrix degeneration, and neovascularization [17].

Hypercholesterolemia is considered as the chief inducer of AS, as increasing of circulating cholesterol increase the endothelial permeability and deposition of lipid particles in the vascular endothelium [18, 19]. Lipid particles primarily LDL in the sub-endothelial space act as chemo-attractants for the monocytes which converted to foamy macrophages. Furthermore, oxidized LDL in the sub-endothelial space activates the expression of scavenger receptors on the macrophages with additional buildup of intracellular cholesterol. These pathological changes encourage plaque formation, narrowing of vascular lumen and development of AS [20]. Atherosclerotic plaques are vulnerable for erosion, rupture, and calcification with nodule formation, and higher infiltration of T cells into atherosclerotic plaque increases vulnerability for rupture and thrombosis [21].

High LDL and TG with low HDL act as strong predictors for the development of premature AS [22]. However, high HDL level is considered as a protective factor against development and progression of AS [23]. Moreover, hypertriglyceridemia is regarded as independent risk factor for progression of AS [24]. Similarly, lipoprotein disorders are associated with AS pathophysiology, increased lipoprotein A is related with AS development [25].

Particularly, macrophage is the most immune cell intricate with progression of AS and atherosclerotic complications including erosion and rupture, typically, immune cells mostly macrophages consume oxidized LDL (ox-LDL) with production of ROS [26]. In order, excessive production of ROS promotes development of oxidative stress and progression of plague instability [27]. Consequently, ox-LDL accelerates macrophage oxidative stress process with progression of oxidative stress.

Oxidative stress together with inflammation enhance AS progression in a vicious cycle as inflammation induces oxidative stress and vice versa. Oxidative stress stimulates the expression of inflammatory signaling pathway, pro-inflammatory cytokines, and chemokines which in turn enhance ROS generation [28]. NADPH-oxidase is extensively expressing enzyme primarily vascular smooth muscle involved in ROS generation, higher expression of NADPH-oxidase is increased by aging process leading to endothelial dysfunction, vascular inflammation, and mitochondrial and cellular-induced oxidative stress [29].

It has been revealed that ox-LDL activates infiltration of monocytes and migration of smooth muscle cells, it contributes to atherothrombosis through induction apoptosis of endothelial cells, plaque erosion, production of tissue factors, and impairment of endogenous anticoagulant pathway [30]. HDL attenuates the production and effect of ox-LDL, though; oxidized HDL (ox-HDL) loss the vasculoprotective effect and act as pro-inflammatory and proartherogenic mediator and increase risk of AS progression [31]. Remarkably, ox-HDL promotes the progression of atherosclerotic plaque erosion and rupture. Hence, ox-HDL is regarded as a potential risk factor for AS and atherothrombosis [31].

**Fig. 2:**
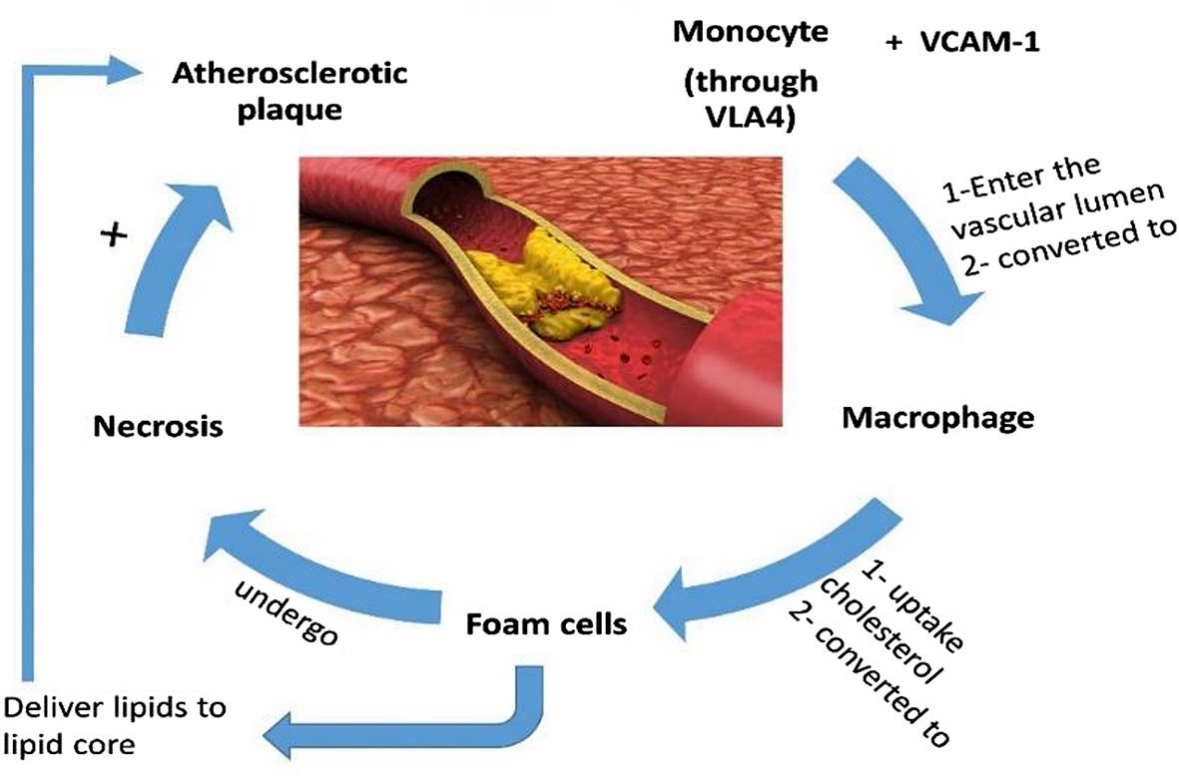
Pathophysiology of atherosclerosis: Monocyte via very late antigen 4 (VLA4) binds vascular cell adhesion molecule 1 (VCAM-1) and enter the vascular lumen, then converted to macrophage which uptake cholesterol and converted to foam cells that undergo necrosis and deliver lipid into the lipid core with formation of atherosclerotic plaque

These observations revealed that AS pathogenesis is a complex process linked to dyslipidemia and associated inflammatory disorders and oxidative stress (Fig. 2).

## Role of vinpocetine in atherosclerosis

### Vinpocetine and inflammation

AS is regarded as an inflammatory disease, atherosclerotic plaque acts as a pathogen associated molecular pattern (PAMP) provokes immune response and infiltration of inflammatory cells [32]. Endothelial injury by ox-LDL promotes the expression of adhesion molecules like E and P selectin that increase recruitment of monocytes and the release of pro-inflammatory cytokines [32]. In addition, PAMP promotes the activation of NF-κB which is a master regulator of immune response, leading to the expression and release of pro-inflammatory cytokines [33]. Therefore, inhibition of NF-κB prevents the expression of adhesion molecules, release of pro-inflammatory cytokines and the development of inflammatory reactions in AS. Experimental studies illustrated that inhibition of NF-κB signaling pathway abrogates the development of AS in mice [34]. VPN has anti-inflammatory effect by inhibiting expression of NF-κB through stabilization inhibitor of IκBα [35]. Zhuang et al. showed that VPN inhibits AS progression in mice through suppression of NF-κB pathway. Importantly, ox-LDL activates NF-κB via stimulation of IKKα/β, IκBα, Akt and PI3K/Akt signaling pathway. VPN inhibits IKKα/β and IκBα preventing NF-κB activation and inflammatory reactions by targeting of IKKα/β is the main pathway for the anti-inflammatory effect of VPN [36]. Interestingly, VPN inhibits NF-κB–dependent inflammatory responses by directly targeting of IKK. VPN constrains intracellular IKK kinase activation and NF-κB–dependent transcriptional activity. VPN inhibits NF-κB–dependent inflammatory responses by directly targeting IKK, independent of its well-known action on PDE-1 activity and Ca^2+^ regulation [37].

Migration and transformation of monocytes to foam ells are mediated by NF-κB and monocyte chemoattractant protein 1 (MCP-1) which promotes the expression of scavenger receptor and differentiation of monocytes to foam cells [38]. Accumulation of ox-LDL in the monocytes triggers the differentiation of monocytes to macrophage and foam cells. MCP-1 increases the expression of CD36 which promote trans-endothelial migration of monocytes [39]. Different studies highlighted that NF-κB increases the expression of MCP-1 in various inflammatory disorders. Therefore, inhibition of NF-κB by VPN may reduce differentiation of monocytes to macrophage and foam cells.

Inflammation is a key factor at all stages of AS progression. Cells involved in pathogenesis of AS are activated by soluble factors and cytokines that influence the development of AS. Pro-inflammatory cytokines accelerate AS progression, while the anti-inflammatory cytokines ameliorate the pathogenesis of AS [40]. Certainly, cytokines are produced by and act (often synergistically) on almost all cells intricate in the pathogenesis of AS, contributing in all steps of the process, from the early endothelial dysfunction to the late formation and disruption of a vulnerable plaque [41]. Pro-atherogenic cytokines such as tumor necrosis factor-alpha (TNF-α), interleukin (IL)-1, and IL-6 are secreted by macrophages, lymphocytes, natural killer cells, and vascular smooth muscle cells [42]. TNF-α and IL-1 signaling is mostly mediated by the p38 mitogen-activated protein kinase (p38MAPK)/NF-κΒ pathway which affects almost all cells involved in atherogenesis by promoting the expression of cytokines, adhesion molecules, and the migration and mitogenesis of vascular smooth muscle and endothelial cells [41]. Remarkably, most of pro-inflammatory cytokines released from macrophages are inhibited by action of VPN via NF-κB-dependent mechanism. VPN blocks monocyte adhesion and migration by inhibiting the expression and action of MCP-1 [43]. Though, VPN exhibits a selective inhibitory activity on the PDE-1 enzyme, though; the anti-inflammatory effect of VPN seems to be PDE-1-independent. VPN inhibits LPS-induced lung inflammation in mice by targeting NF-κB activation and, consequently, the production of NF-κB-related cytokines TNF-α and IL-1β, as well as the recruitment of polymorph nuclear cells [37]. VPN has anti-atherogenic effect through inhibition of NF-κB activation resulting in the inhibition the production of inflammatory cytokines like TNF-α and IL-6, and other inflammatory mediators in a PDE-1-independent manner [36]. In a model of endotoxemia, intraperitoneal administration of VPN reduced the expression of IL-1β and TNF-α in the hippocampus [44]. VPN has an analgesic activity in acetic acid-induced visceral nociception, validating the possible use of this compound in inflammatory pain conditions [45]. Nevertheless, it was not determined if similar analgesic and anti-inflammatory effects could be obtained by oral treatment with VPN in LPS-induced pain [45]. Besides, protective effect of VPN against ischemic reperfusion injury (IRI) could be attributed to inhibtion of NADPH oxidase/Nrf2, IKKβ/NF-κB p65, and cleaved caspase-3 expressions. Thus, VPN could improve oxidant/antioxidant balance, suppress inflammatory response, and promote cell survival after IRI [46]. These verdicts suggest that VPN may be effective against the development of inflammatory disorders in AS by suppressing of inflammatory reactions.

### Vinpocetine and plaque stability

Most of acute cardiovascular events are due to thrombotic occlusion caused by ruptured atherosclerotic plaques. Plaque rupture is frequently caused by an inflammatory degradation of the plaque connective tissue, most importantly the fibrous cap [30]. Atherosclerotic plaques that are at high risk of rupturing are often referred to as vulnerable plaques. Such plaques are characterized by abundant inflammation, a large core of lipids and necrotic cells, and a thin fibrous cap. The plasma level of certain pro-inflammatory cytokines can be used as surrogate markers for the identification of patients with high-risk plaques [30]. Thus, abundant cytokines within the atherosclerotic plaques may diffuse into the circulation, and thereby; plasma levels of such cytokines could reflect the inflammatory activity in the plaques. Plasma levels of several cytokines have been shown to correlate with the progression of the AS or have been considered as markers of cardiovascular events [30].

It has been shown that macrophages within the atherosclerotic plaque are regarded as the major source of pro-inflammatory and inflammatory cytokines. Macrophage is considered as a key regulator of metabolic signals and inflammatory response in atherosclerotic plaque formation [47]. Therefore, macrophage activity and plaque contents are change in a dynamic balance. Macrophage lipid contents triggers inflammation and immune response by augmentation the sensitivity of TLR-4 to their ligands by inducing expression of nod-like receptor pyrin 3 (NLRP3) inflammasome [48]. Of note, the interaction of ox-LDL with monocytes/macrophages in the atherosclerotic plaque promotes inflammatory and oxidative stress disorders [47]. It has been shown that VPN attenuates the formation of atherosclerotic lesion in ApoE knockout mice. Besides, in vitro study illustrated that VPN blocks the uptake of ox-LDL in cultured macrophage through inhibition the expression of ox-LDL receptor [49]. VPN has weak inhibitory effect on the formation of foam cells, though it has strong inhibitory effect on ROS generation and the expression of pro-inflammatory cytokines in macrophages of atherosclerotic plaque as it attenuates the uptake of ox-LDL by foam cells [36].

Furthermore, VPN suppresses carotid intimal hyperplasia through inhibition the migration and proliferation of vascular smooth muscle cells (VSMCs) [50]. Wang et al. showed that VPN reduces the progression of carotid neo-intimal hyperplasia in diabetic rat following balloon injury [51]. Migration and proliferation of VSMCs are regulated by platelet derived growth factor (PDGF) which secreted from endothelial cells. PDGF promotes VSMCs phenotype shift during vascular injury by regulating autophagy [52]. Likewise, migration and proliferation of VSMCs are also augmented by Akt and extracellular signal regulated protein kinase 1/2 (ERK1/2) via activation of different signaling pathway [53]. In vitro study demonstrated that VPN blocks PDGF and ERK1/2-mediated VSMCs [50]. These observations suggest that VPN has a protective effect against intimae hyperplasia and vascular remodeling during development of AS.

Moreover, VPN stabilizes atherosclerotic plaque by increasing collagen content, plaque cap thickness and decreasing of lipid-rich core size through attenuation the expression of MMP-9 and TNF-α [36]. MMP-9 induces the degradation of plaque matrix causing plaque necrosis, rupture, and thrombosis. Inhibition of MMP-9 by the effect of VPN promotes plaque stability and prevents plaque-mediated complications. Thus, VPN may play a crucial role in preventing the development of atherosclerotic complication by enhancing of plaque stability.

### Vinpocetine and PDE in atherosclerosis

It has been shown that PDE-1 A is highly expressed in foam cells of atherosclerotic plaque, while; PDE-1B and PDE-1 C are highly abundant in VSMCs. Different studies illustrated that PDE inhibitors by increasing cAMP/cGMP in VSMCs reduce intima media thickness and the progression of AS [54]. A prospective, randomized study involved 329 diabetic showed that cilotazol reduced intima media thickness compare to aspirin [55]. As well, sildenafil (PDE-5 inhibitor) improves endothelial dysfunction and vascular injury in mice [54]. VPN is a selective PDE-1 inhibitor increases cAMP/cGMP in VSMCs with subsequent inhibition of migration and differentiation. Intracellular cAMP is regulated by PDE and adenylyl cyclase that control atherogenesis through modulation the recruitment and migration of monocytes, and differentiation of macrophages to foam cells [56]. It has been shown that cAMP inhibits the release of pro-inflammatory cytokines from differentiated macrophage in the atherosclerotic plaque. Besides, cAMP attenuates the progression of atherosclerotic plaque by reducing macrophage cholesterol content via the expression of macrophage secretory pathway. In addition, increasing of cAMP is associated with inhibition the expression of MMP-9, platelet activation, thrombosis and atherogenesis [57]. Likewise, cGMP plays a critical role in maintaining of endothelial function through induction the expression and the release of NO. It has been observed that alteration of cGMP/NO signaling pathway is associated with atherogenesis and the development of AS. A previous experimental study demonstrated that chronic hypercholesterolemia-induced ROS reduce the availability of vasoprotective NO in rabbit, this effect was mediated by reducing of cGMP which intricate in intima proliferation and endothelial dysfunction through regulation of survival and phenotypic plasticity of VSMCs during vascular injury and the development of AS [58]. VSMCs express different types of cGMP effectors like cGMP-dependent protein kinase and NO-sensitive guanylyl cyclase that involved in the inhibition of VSMCs proliferation. Therefore, cGMP has a major role in regulating of atherogenesis by inhibiting VSMCs proliferation [59]. Thus, VPN by increasing of cAMP/cGMP could be effective in the management of AS through maintaining the endothelial integrity and inhibition of VSMCs proliferation [60].

### Vinpocetine and oxidative stress in atherosclerosis and endothelial dysfunction

Oxidative stress is involved in the pathogenesis of various cardiovascular diseases including AS. AS represents a state of oxidative stress characterized by protein and lipid oxidations in the vascular endothelium [27]. Overproduction of ROS is an integral pathway in the development and progression of endothelial dysfunction and AS [61]. Oxidative stress-induced endothelial dysfunction is mediated by depletion of endothelium NO [62, 63]. It has been illustrated that oxidative stress promotes the formation of atherosclerotic plaque through induction the expression of adhesion molecules, inflammation and the development of endothelial dysfunction [64]. Endothelial NADPH oxidase is a master enzyme for generation of ROS that correlate with progression of endothelial dysfunction and AS [65]. Different studies revealed that LDL directly activates the endothelial NADPH oxidase through the expression of signal transduction like phospholipase A2 and release of arachidonic acid (AA) which involved in activation of NADPH oxidase [66, 67]. Monocytes, macrophages, VSMCs, and endothelial cells have ability to oxidized LDL through NADPH oxidase-dependent pathway [68]. Moreover, ROS are also produced by other enzymes and pathways including xanthin oxidase, mitochondrial eNOS and uncoupling eNOS [65]. The vascular endothelium is protected from the effect of oxidative stress by antioxidant enzyme system including catalase, superoxide dismutase, paraoxonase and glutathione peroxidase [69]. ROS induces atherogenesis by oxidative modification of phospholipids and lipoproteins. Therefore, oxidative/antioxidant imbalance promote macrophage polarization, formation of foam cells and formation of atherosclerotic plaque [20, 70].

VPN is regarded as an antioxidant agent reduces the propagation of oxidative stress by inhibiting the generation of ROS, inflammatory and oxidative stress disorders [5, 71]. In response to LPS, both macrophages and neutrophils through TLR4/NF-κB produce large amount of mediators including ROS and pro-inflammatory cytokines leading to inflammation and associated oxidative stress [71]. An experimental study revealed that administration of VPN 30 mg/kg inhibits LPS-induced oxidative stress in mice through inhibition of NF-κB signaling. Al-kuraishy et al. revealed that VPN attenuates gentamicin-induced acute kidney injury in rats by inhibiting the development and progression of oxidative stress [5]. Recently, VPN-induced inhibition of PDE1 prevents brain oxidative stress in behavioral phenotype of autism spectrum disorders [72]. As well, VPN mitigates the inflammatory and oxidative stress disorders in Covid-19 which linked with hyperinflammation and oxidative stress [3]. These findings proposed that VPN could be effective in reducing of oxidative stress a cornerstone in the pathogenesis of AS through inhibition of NF-κB and PDE1.

**Fig. 3:**
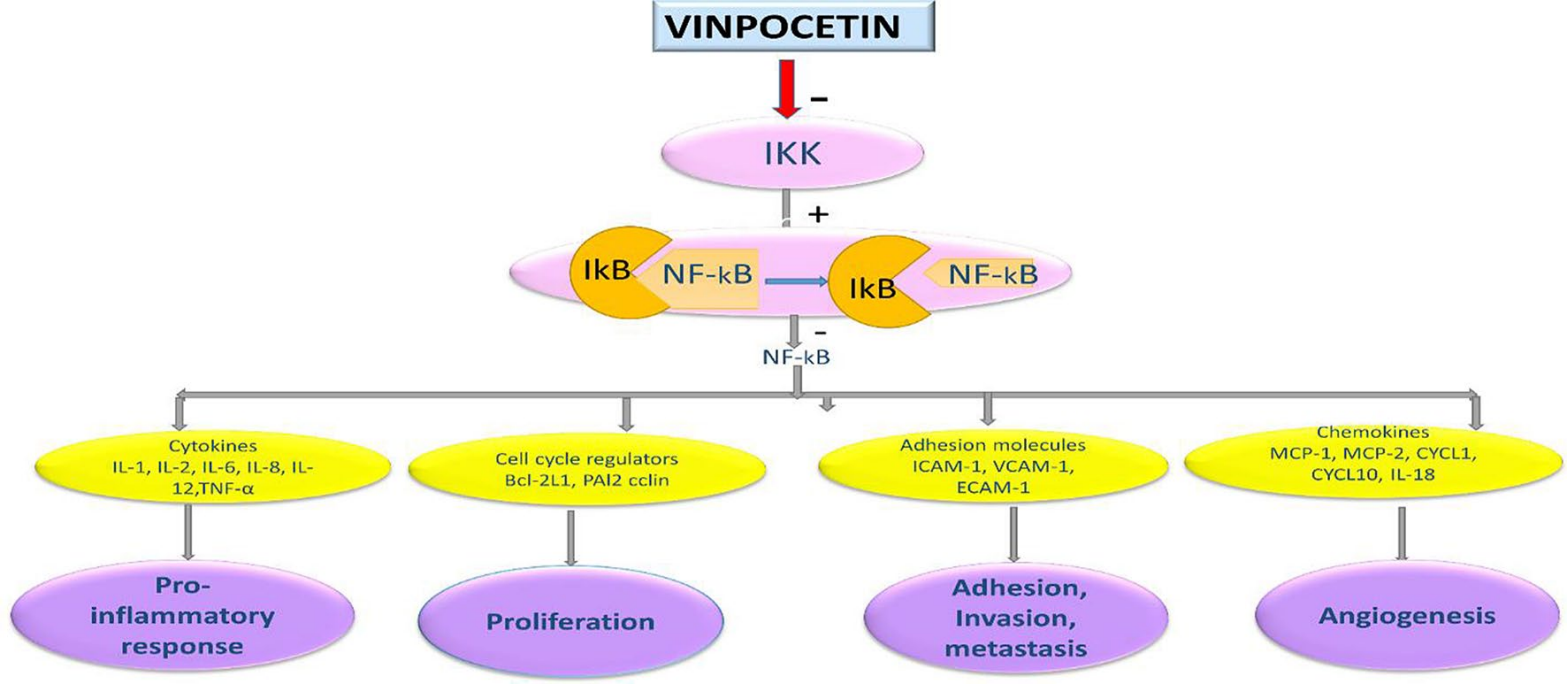
Role of vinpocetine in atherosclerosis: Vinpocetine inhibits NF-κB and related signaling leading suppression release of pro-inflammatory cytokines, reactive oxygen species (ROS), proliferation, and migration of vascular smooth muscle cells

Furthermore, hemodynamic shear stress is a frictional force on the vascular endothelium controls endothelium homeostasis in normal physiological process [73]. The laminar shear stress prevents endothelial injury and the development of AS, whereas; disturbed blood flow induces the development of atherothrmbosis [74]. It has been reported that disturbed blood flow due to endothelial dysfunction in AS induce activation of endothelial voltage-gated Na^+ 2^ channel which promote expression of ERK1/2 and NF-κB activation [75]. Of note, VPN is regarded as a potent inhibitor of voltage-gated Na^+ 2^ channel preventing cell toxicity and death through mitigation of ERK1/2 and NF-κB activation [8]. Taken together, VPN through mitigation of inflammatory and oxidative stress, and plaque stability effects could be effective agent in the management of AS (Fig. 3).

## Conclusion

VPN is a potent inhibitor of PDE-1 that has anti-inflammatory and antioxidant effects through inhibition the expression of NF-κB. VPN has been shown to be effective against the development and progression of AS. Most of pro-inflammatory cytokines released from macrophages are inhibited by action of VPN through NF-κB-dependent mechanism. VPN prevents NF-κB activation and inflammatory reactions. Besides, VPN blocks monocyte adhesion and migration by inhibiting the expression of pro-inflammatory cytokines. VPN may reduce the differentiation of monocytes to macrophage and foam cells. Remarkably, most of pro-inflammatory cytokines released from macrophages are inhibited by action of VPN via NF-κB-dependent mechanism .VPN blocks monocyte adhesion and migration by inhibiting the expression and action of MCP-1. In addition, VPN may play a crucial role in preventing development of atherosclerotic complication through enhancement of plaque stability. VPN by increasing of cAMP/cGMP could be effective in the management of AS by maintaining endothelial integrity and inhibition of VSMCs proliferation. VPN is effective in reducing of oxidative stress a cornerstone in the pathogenesis of AS through inhibition of NF-κB and PDE1. Taken together, VPN promotes plaque stability and prevents erosion and rupture of atherosclerotic plaque. In conclusion, VPN through mitigation of inflammatory and oxidative stress with plaque stability effects could be effective agent in the management of AS. This review cannot gives the final conclusion regarding the atheroprotective role of VPN in AS. Herein, preclinical and clinical studies are reasonable in this regard.

## Data Availability

All data generated or analyzed during this study are included in this published article.
